# Editorial: Advances in targeted therapy and biomarker research for endocrine-related cancers, volume II

**DOI:** 10.3389/fendo.2025.1736682

**Published:** 2025-11-17

**Authors:** Mei Guo, Chun Zhang, Yang Wu, Xudong Cheng, Yanshuang Zhuang, Zili Zhang

**Affiliations:** 1 School of Nursing, Nanjing University of Chinese Medicine, Nanjing, China; 2 Affiliated Hospital of Nanjing University of Chinese Medicine, Nanjing University of Chinese Medicine, Nanjing, China; 3 Pancreas Center, the First Affiliated Hospital of Nanjing Medical University, Nanjing, China; 4 Suzhou TCM Hospital Affiliated to Nanjing University of Chinese Medicine, Suzhou, China; 5 Department of Science and Technology, Taizhou Affiliated Hospital of Nanjing University of Chinese Medicine, Taizhou, China; 6 School of Pharmacy, Nanjing University of Chinese Medicine, Nanjing, China

**Keywords:** endocrine-related cancers, targeted therapy, biomarker research, clinical application, personalized treatment

The domain of endocrine-associated neoplasms is currently witnessing a dynamic evolutionary trajectory, propelled by considerable advancements in the elucidation of the molecular and genetic architecture underlying these pathologies (1). These cancers, spanning a diverse array of endocrine system tumors, pose distinctive clinical challenges owing to their complex interplay with hormonal regulatory networks (2). Recent decades have witnessed an explosion in targeted therapeutic development, fostering renewed hope for precision-guided therapeutic strategies tailored to individual patient profiles (3). Complementing this progress, the discovery and rigorous validation of clinically actionable biomarkers have become pivotal for enhancing diagnostic accuracy, refining prognostic risk stratification, and optimizing treatment selection (4). In parallel, groundbreaking technological innovations such as patient-derived organoid cultures and microphysiological organ-on-a-chip platforms have emerged as transformative tools, offering more physiologically authentic model systems to investigate tumor pathophysiology and pharmacodynamic responses (5). These advancements collectively underscore the critical role of translational biomedicine in accelerating the clinical translation of basic research discoveries, thereby creating novel opportunities to advance understanding and therapeutic management of endocrine-associated cancers.

The objective of this Research Topic was to advance pioneering investigations and strengthen our comprehension of endocrine-related cancers. Through emphasizing seminal studies on targeted therapies and biomarker researches, we aim to bridge the translational gap between preclinical discovery and clinical implementation. By establishing an interdisciplinary collaborative platform, we endeavor not only to disseminate state-of-the-art scientific findings but also to catalyze the development of innovative therapeutic approaches. We integrate basic research insights with clinical application strategies, thereby optimizing translational pathways to improve therapeutic outcomes for patients with endocrine oncological disorders. A series of articles have been published under this Research Topic, each contributing to the synthesis of knowledge and the advancement of precision oncology in this specialized domain.

Endocrine neoplasms represent a multifarious neoplastic cohort characterized by distinct biological phenotypes (6). Although predominantly slow-growing, this group encompasses highly aggressive variants, and any detectable metastatic indicator significantly correlates with elevated recurrence risk and reduced responsiveness to standard therapeutic regimens (7). Chimeric antigen receptor (CAR)-T cell therapy, an innovative oncology breakthrough, shows potential for endocrine malignancies but exhibits suboptimal clinical outcomes, prompting exploration of novel strategies (8). Wang et al. summarized recent advances in identifying targets of endocrine cancer for CAR therapy. They showed that promising antigens for CAR-T cell targeting of endocrine cancer include delta-like ligand 3, somatostatin receptor, B7-H3, gangliosides, glycoprotein, aberrant glycosylation, and miscellaneous targets. Multi-antigen targeting diminishes antigen escape risks from loss or mutation, ensuring sustained immune activation in CAR cell therapies. Additionally, CAR-related endocrine tumor therapy has been extended from T cells to NK cells and macrophages. NK cells utilize MHC-independent recognition mechanisms, minimizing allotransplantation risks. Their distinct cytokine profile reduces cytokine storm likelihood compared to T cells, while limited survival time correlates with lower off-target toxicity. These cellular platforms offer improved safety profiles and may enhance efficacy in solid tumor contexts, though further validation is needed to optimize dosing and persistence.

Thyroid carcinoma is the most frequently diagnosed endocrine neoplasm, and presents substantial therapeutic dilemmas owing to its heterogeneous biological properties (9). Growth differentiation factor 15 (GDF15), a stress-induced cytokine, has been implicated in tumor progression and senescence modulation across multiple malignancies (10). However, its precise role in thyroid cancer pathogenesis, particularly its interplay with the p53 signaling axis, remains incompletely characterized. Therefore, Ma et al. elucidated the functional involvement of GDF15 in thyroid cancer progression and its regulatory mechanisms governing cancer cell senescence. Methodologically, they integrated analysis of publicly available datasets with clinical specimens to assess GDF15 expression profiles and clinical correlations. *In vitro* models were constructed using human thyroid carcinoma cell lines, with GDF15 expression modulated via siRNA-mediated knockdown. Molecular validation employed RT-qPCR and immunoblotting to quantify target gene expression. Functional assessments included proliferation assays, migratory/invasive capacity evaluation, and senescence characterization. Mechanistic insights were gained through RNA-seq and pathway enrichment analysis, focusing on p53 signaling and senescence regulation. They found GDF15 overexpression in thyroid carcinoma tissues compared to adjacent normal tissue, correlating with lymphatic metastasis status. GDF15 silencing suppressed proliferative, migratory, and invasive phenotypes while inducing senescence markers. Transcriptomic profiling revealed p53 protein upregulation following GDF15 depletion. Rescue experiments confirmed p53-mediated reversal of senescence phenotypes. These findings establish GDF15 as a dual regulator of thyroid cancer progression and senescence through p53-dependent mechanisms, highlighting its potential as both a therapeutic target and prognostic biomarker.

Adrenocortical carcinoma (ACC) metastasis constitutes a major clinical hurdle, with limited mechanistic understanding and scarce effective therapeutic options (11). Thus, discovering new molecular targets and more effective drugs for metastatic ACC is essential. In recent decades, genome-wide association analyses have revolutionized our comprehension of cancer pathogenesis and therapeutic innovation (12). Weighted gene co-expression network analysis (WGCNA) emerges as a robust bioinformatics framework, enabling the construction of gene co-expression modules characterized by module eigengenes and intramodular hub genes (12). This methodology facilitates the identification of functionally correlated gene clusters and key regulatory nodes, providing systematic insights into molecular mechanisms underlying disease progression and potential therapeutic targets (13). Kong et al. applied WGCNA to RNA-seq data of 73 ACC cases from TCGA database and selected the most significant module concerned with metastasis. Through rigorous bioinformatic filtering, seven central genes including ZWINT, CDK1, BIRC5, CCNA2, CCNB1, TYMS, and TPX2 were identified as pivotal regulators in ACC metastasis. These genes exhibit strong prognostic value and may serve as robust biomarkers for metastatic stratification. Therapeutic agents targeting these molecular nodes could offer novel, low-toxicity interventions for metastatic ACC management. These findings provide a foundation for further mechanistic exploration and clinical translation, potentially advancing precision oncology strategies in this aggressive malignancy.

Neuroendocrine neoplasms (NENs) represent uncommon malignancies arising from endocrine-derived cells, primarily localized within the gastroenteropancreatic system and pulmonary parenchyma (14). Metastatic NETs patients frequently exhibit prolonged survival, necessitating careful balancing of treatment efficacy and toxicity management to avoid persistent adverse effects, while their unique clinical and biological features underscore the critical role of precise classification, molecular biomarker monitoring, and personalized therapeutic strategies in optimizing patient outcomes (15). Peptide receptor radionuclide therapy (PRRT) is an effective and well-tolerated treatment for advanced NETs. However, persistent thrombocytopenia (PT) has been reported and may compromise further therapies and outcomes (16). Therefore, Ferrara et al. explored potential predictive factors for PT in a larger population of 47 metastatic NET patients. They found that 17% of PT incidence correlated with relatively high bone metastatic burden and spleen length. Physicians should be vigilant in the event of a significant drop in platelet count after the first cycle of PRRT. They highlighted the necessity to detect early hematologic toxicity markers in PRRT patients. Numerical analysis of bone marrow involvement and initial platelet count drops across treatment cycles may aid in predicting hematopoietic damage. Splenic dimensions could act as a novel, accessible biomarker for hematologic risk. Additional validation in larger cohorts using prospective design and dosimetric integration is essential to confirm these factors’ predictive value and refine individualized treatment models.

In conclusion, these articles within this Research Topic present a systematic synthesis of targeted therapeutic strategies and molecular biomarker exploration for endocrine-related neoplasms. By scrutinizing the molecular and genetic architecture of these tumors, these studies yield pivotal insights that advance diagnostic accuracy, prognostic stratification, and personalized treatment paradigms. The discovery and validation of specific molecular biomarkers hold promise for enhancing early detection capabilities and enabling precise, patient-specific therapeutic guidance. Furthermore, the development of targeted therapies represents an innovative approach to improving clinical outcomes. These contributions shape emerging clinical frameworks for endocrine malignancy management. Lastly, we thank all our contributors who enriched this Research Topic by submitting manuscripts highlighting their highly valuable and interesting research studies.

## References

[1] Feijó M Carvalho TMA Fonseca LRS Vaz CV Pereira BJ Cavaco JEB . Endocrine-disrupting chemicals as prostate carcinogens. Nat Rev Urol. (2025) 22:609–31. doi: 10.1038/s41585-025-01031-9, PMID: 40379948

[2] Revilla G Cedó L Tondo M Moral A Pérez JI Corcoy R . LDL, HDL and endocrine-related cancer: From pathogenic mechanisms to therapies. Semin Cancer Biol. (2021) 73:134–57. doi: 10.1016/j.semcancer.2020.11.012, PMID: 33249202

[3] Akyuz EM Gultekin M Ramage JM Spendlove I Jackson AM Adhikaree J . Epigenetic regulation of immune responses in endocrine-related cancers and its role in immunotherapy. Cancers (Basel). (2025) 17:3342. doi: 10.3390/cancers17203342, PMID: 41154396 PMC12563070

[4] Wu Y Zhang Z . Editorial: Advances in targeted therapy and biomarker research for endocrine-related cancers. Front Endocrinol (Lausanne). (2024) 15:1533623. doi: 10.3389/fendo.2024.1533623, PMID: 39722807 PMC11668659

[5] Rodríguez-Rodero S Delgado-Álvarez E Fernández AF Fernández-Morera JL Menéndez-Torre E Fraga MF . Epigenetic alterations in endocrine-related cancer. Endocr Relat Cancer. (2014) 21:R319–330. doi: 10.1530/ERC-13-0070, PMID: 24898948

[6] Xu F Shi J Qin X Zheng Z Chen M Lin Z . Hormone-glutamine metabolism: A critical regulatory axis in endocrine-related cancers. Int J Mol Sci. (2022) 23:10086. doi: 10.3390/ijms231710086, PMID: 36077501 PMC9456462

[7] León-González AJ Jiménez-Vacas JM Fuentes-Fayos AC Sarmento-Cabral A Herrera-Martínez AD Gahete MD . Role of metformin and other metabolic drugs in the prevention and therapy of endocrine-related cancers. Curr Opin Pharmacol. (2021) 60:17–26. doi: 10.1016/j.coph.2021.06.002, PMID: 34311387

[8] Du X Goh PK Ma C Coughlan E Greatorex S Porter LH . Targeting PTPN2 enhances human CAR T cell efficacy and the development of long-term memory in mouse xenograft models. Sci Transl Med. (2025) 17:eadk0627. doi: 10.1126/scitranslmed.adk0627, PMID: 41160667

[9] Zambelli V Orlando G Fornaro M Vocino Trucco G Rapa I Napoli F . High prevalence of potential molecular therapeutic targets in poorly differentiated thyroid carcinoma. Endocr Pathol. (2025) 36:38. doi: 10.1007/s12022-025-09883-y, PMID: 41123735 PMC12546271

[10] Khetarpal SA Li H Vitale T Rhee J Challa S Castro C . Cardiac adaptation to endurance exercise training requires suppression of GDF15 via PGC-1α. Nat Cardiovasc Res. (2025) 4:1277–94. doi: 10.1038/s44161-025-00712-3, PMID: 40993371 PMC13158892

[11] Kuhlen M Schmutz M Kunstreich M Redlich A Claus R . Targeting pediatric adrenocortical carcinoma: Molecular insights and emerging therapeutic strategies. Cancer Treat Rev. (2025) 136:102942. doi: 10.1016/j.ctrv.2025.102942, PMID: 40258305

[12] Zhang M Zhang Y Deng Z Liu T Liang Y Li Q . GWAS and gene co-expression network analysis reveal the genetic control of seed germination under salt stress in maize. Theor Appl Genet. (2025) 138:285. doi: 10.1007/s00122-025-05077-6, PMID: 41148340

[13] Yang H Pang S Guo S Wang Y Kong M Zhu P . Identification of key genes associated with overwintering in Histia rhodope larva using gene co-expression network analysis. BMC Genomics. (2025) 26:923. doi: 10.1186/s12864-025-12136-1, PMID: 41094399 PMC12522936

[14] Goeppert B Charbel A Toth R Zhang Y Tabbakh D Albrecht T . DNA methylation patterns facilitate tracing the origin of neuroendocrine neoplasms. Nat Commun. (2025) 16:9477. doi: 10.1038/s41467-025-65227-8, PMID: 41145514 PMC12559432

[15] Panzuto F Barbi S Trama A Fazio N . The importance of education and training in neuroendocrine neoplasms: challenges and opportunities for multidisciplinary management. Cancer Treat Rev. (2025) 139:102998. doi: 10.1016/j.ctrv.2025 40714368

[16] Xue B Yan L Ye M Gu D Qian J He N . PROTAC-Surufatinib suppresses pancreatic neuroendocrine neoplasms progression by inducing ferroptosis through inhibiting WNT/β-catenin pathway mediated by HMOX1. Int J Biol Sci. (2025) 21:2476–92. doi: 10.7150/ijbs.106357, PMID: 40303295 PMC12035883

